# Influence of Monochromatic Light during Incubation on the Production and Metabolism of Low-Temperature Broiler Chicks

**DOI:** 10.3390/ani14111620

**Published:** 2024-05-30

**Authors:** Stéfane Alves Sampaio, Rodrigo Fortunato de Oliveira, Kelly Fernanda Borges, Alison Batista Vieira Silva Gouveia, Julia Marixara Sousa da Silva, Adelir José Santos, Murilo Sousa Carrijo, Fabiana Ramos dos Santos, Francisco Ribeiro de Araújo Neto, Ana Paula Cardoso Gomide, Cibele Silva Minafra

**Affiliations:** Goiano Federal Institute of Education Science and Technology (Instituto Federal Goiano—IF Goiano), Rio Verde 75.901-970, GO, Brazil; stefanesamp@gmail.com (S.A.S.); fortunatorodrigo@ymail.com (R.F.d.O.); kellyfernandaborges2512@gmail.com (K.F.B.); alisonmestre28@gmail.com (A.B.V.S.G.); marixaraj@gmail.com (J.M.S.d.S.); adelir.jsantos@gmail.com (A.J.S.); carrijoms@hotmail.com (M.S.C.); fabiana.santos@ifgoiano.edu.br (F.R.d.S.); francisco.neto@ifgoiano.edu.br (F.R.d.A.N.); ana.gomide@ifgoiano.edu.br (A.P.C.G.)

**Keywords:** development embryonic, infrared camera, thermal stress, LED

## Abstract

**Simple Summary:**

Simple Summary: Artificial incubation is essential in the development of poultry production, since it represents the beginning of the production process and the key point in avoiding problems in post-hatching chicks. Humidity and air temperature during the incubation period are essential elements for good embryonic development. Lighting is one of the main environmental elements in control of several physiological and behavioral processes of birds; therefore, it allows the establishment of rhythmicity, the synchronization of several indispensable functions of their body and the stimulation of secretion of several hormones related to growth, maturation and reproduction of birds. LED lamps can produce lights that change color, intensity and distribution, providing an environment closer to the natural one for birds, as it ensures better expression of their behavior.

**Abstract:**

The use of artificial lighting during the incubation phase is a tool that has been studied with the aim of increasing the production rates and hatchability. Using this, this study aims to investigate the effects of the luminous incidence of white and red monochromatic light on the production and metabolism of broiler chicks subjected to low temperatures. A total of 315 eggs of Ross 708 heavy breeders were used. The eggs were distributed randomly, with 35 eggs per tray, totaling 105 eggs per incubator. The treatments were the following: incubation without the use of light; the use of white monochromatic light; and the use of red monochromatic light. The lamps used were of the LED type. The samples were distributed in the factorial completely randomized experimental design with position effect on the tray. Candling, egg weighing, calculating the probability of survival and egg weight loss were performed. Temperatures were recorded using a thermographic camera. At birth, three chicks per tray were euthanized for evaluation: weight with and without yolk residue, gastrointestinal tract biometry, and blood and liver biochemistry. Analyses were performed using the R computational program. It was observed that there was a significant effect of the treatments on the levels of calcium, phosphorus, cholesterol, amylase, glucose, urea and glutamate pyruvate transaminase on the biochemical profile of the blood and on the thermographic temperatures of the eggs; the experiment was kept at low temperatures resulting in thermal stress, with an average temperature of 34.5 °C. Therefore, the use of red and white monochromatic light in the artificial incubation process for brown-colored eggs is not recommended, because in the post-hatching phase, it promoted the metabolism dysregulation on the blood biochemical profile to control the differentiation in the wavelength of traditional incubation.

## 1. Introduction

Artificial incubation is essential in the development of poultry production, since it represents the beginning of the production process and a key point in avoiding problems in post-hatching chicks [1]. The embryonic period of birds is a very important phase, as it represents a large part of their life; for example, a broiler lives, on average, 42 days and goes through an egg incubation period of 21 days until the hatching, which represents about 30% of the chicken’s life [2].

In addition, incubators have played an important role in the development of the commercial poultry industry, helping to uniformly produce large numbers of day-old chicks with greater hatchability than the natural incubation process [3]. The quality of the day-old chick is strictly related to the characteristics of the incubated egg. In this sense, more research on this stage of the bird’s life should be conducted to seek innovations that favor the production of viable and high-quality chicks [4].

Humidity and air temperature during the incubation period are responsible for driving embryo development and growth [5]. Modern incubators work with fixed temperatures between 37.5 and 37.8 °C, and most of the current lineages present good hatchability rates [6], whose best results are observed under a relative humidity close to 60–65% [7].

Temperature variations below or above the usual can delay or accelerate embryo development [8], changing the duration of incubation. They can compromise the development of the embryo by reducing hatchability, the quality of chicks at hatching, and metabolic changes, which involve higher or lower energy expenses, depending on the incubation temperature [9].

Lighting is one of the main environmental elements in the control of several physiological and behavioral processes of birds; therefore, it allows the establishment of rhythmicity, the synchronization of several indispensable functions of their body and the stimulation of secretion of several hormones related to the growth, maturation and reproduction of birds [10,11,12,13,14].

Light stimulation can accelerate embryonic development, increase hatchability and positively influence the productive performance and muscle development of broiler chickens at the end of the production cycle [15].

LED lamps can produce lights that change color, intensity and distribution, providing an environment closer to the natural one for birds, and ensuring a better expression of their behavior [16,17]. In this sense, the light-emitting diode is an effective technological alternative to maximize production and reduce energy expenses [18].

Thus, the present study aimed to investigate the effects of light incidence of white and red monochromatic lights on the production and metabolism of broiler chicks subjected to low temperatures.

## 2. Materials and Methods

### 2.1. Location and Period of Experiment

The experiment was conducted at the Laboratory of Biochemistry and Animal Metabolism of the Goiano Federal Institute Campus Rio Verde—GO. The research project was approved by the Animal Research Ethics Committee of that institution, under protocol number 9019300919.

### 2.2. Sample Collection and Experimental Procedure

A total of 315 eggs from Ross 708 commercial line heavy matrices (matrices producing brown-colored-shell eggs) were used. All eggs underwent prior visual selection, in which the hatchable eggs were those presenting clean and intact shells and an elliptical shape. Pointed, cracked, broken, dirty or rounded eggs were discarded. After visual selection, all eggs were individually identified and weighed for the obtainment of the average weight at the time of incubation.

A total of three Chocomaster incubators (automatic hatchery model Nani, Manufacturer: HHD, Ndeal Incubator, China), provided with 60% humidity, was used. The eggs were placed under three different lighting conditions: without lighting, with white monochrome lighting, and with red monochrome lighting, with 105 eggs (replicates) in each incubator. Eggs were randomly distributed in the three trays of the incubators, with 35 eggs distributed per tray.

White (400 to 700 nanometers with an average of 65 lux) and red (700 nanometers with an average of 45 lux) LED lamps (model: LED Bulb Ball, Manufacturer/Factory & Trading Company, China) were fixed only on the ceiling of the incubators, providing three distinct positions of the eggs inside the trays in relation to the light source. The samples were distributed in the factorial completely randomized experimental design with position effect on the tray as a factor of the light stimulus. Automatic egg turning was programmed for every hour at a 45° angle, from the 3rd to the 18th day of incubation.

On the 1st, 7th, 14th and 18th day of incubation, candling of all incubated eggs was performed so that infertile eggs or eggs with embryonic mortality were verified and eliminated. All eggs on each shelf were weighed, then placed in the incubator. When the 18 days of incubation were completed, the weight loss of the eggs was evaluated. Percent weight loss was obtained by the following formula, according to Barbosa et al. [19].

$$weight\;loss\;(\%)=\frac{\left(egg\;weight\;at\;incubation—egg\;weight\;at\;18th\;day\times 100\right)}{egg\;weight\;at\;incubation}$$

**Figure 1:**
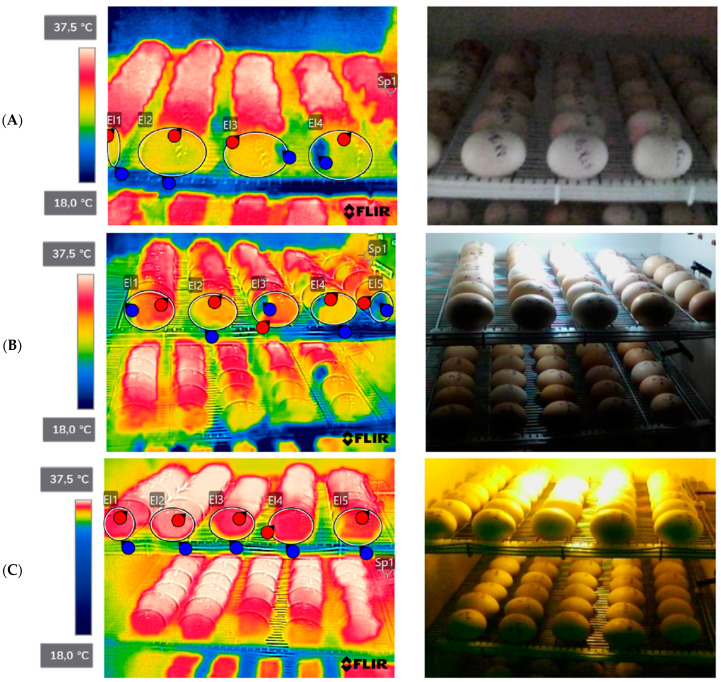
Thermographic images of broiler eggs without lighting and with lighting. (**A**) Treatment without light; (**B**) treatment with white monochromatic light; (**C**) treatment with red monochromatic light. Source: personal archive, 2022.

The surface temperatures of the eggs were recorded in the periods of the 1st, 7th, 14th, 18th and 21st days, using the FLIR C2 thermographic camera, and thermographic images of the entire length of the tray were obtained (Figure 1). Images were captured at a horizontal distance of about 1 m from the eggs. The camera parameters were set to 0.95 emissivity; 27.7 °C for reflected temperature; 27.7 °C for atmospheric temperature; 70.0% for relative humidity; 27.7 °C for external optics temperature; and 1.00 for external optics transfer. Images were processed using the brand’s own software FLIR v. C3-X (FLIR Thermal Studio, Instrutemp, São Paulo, Brazil), from selected points.

The experiment was kept at low temperatures, causing thermal stress, with a maximum temperature range of 35.6 °C and a minimum temperature of 33.6 °C, and a maximum relative humidity of 72% and a minimum relative humidity of 68%.

After birth, three chicks per shelf, totaling 27 chicks, from different positions inside the incubators, were weighed and subsequently desensitized and euthanized by jugular vein bleeding and weighed without yolk residue. Body weight with and without yolk residue was expressed in absolute values and values relative to egg weight (chick weight × 100/egg weight).

**Figure 2:**
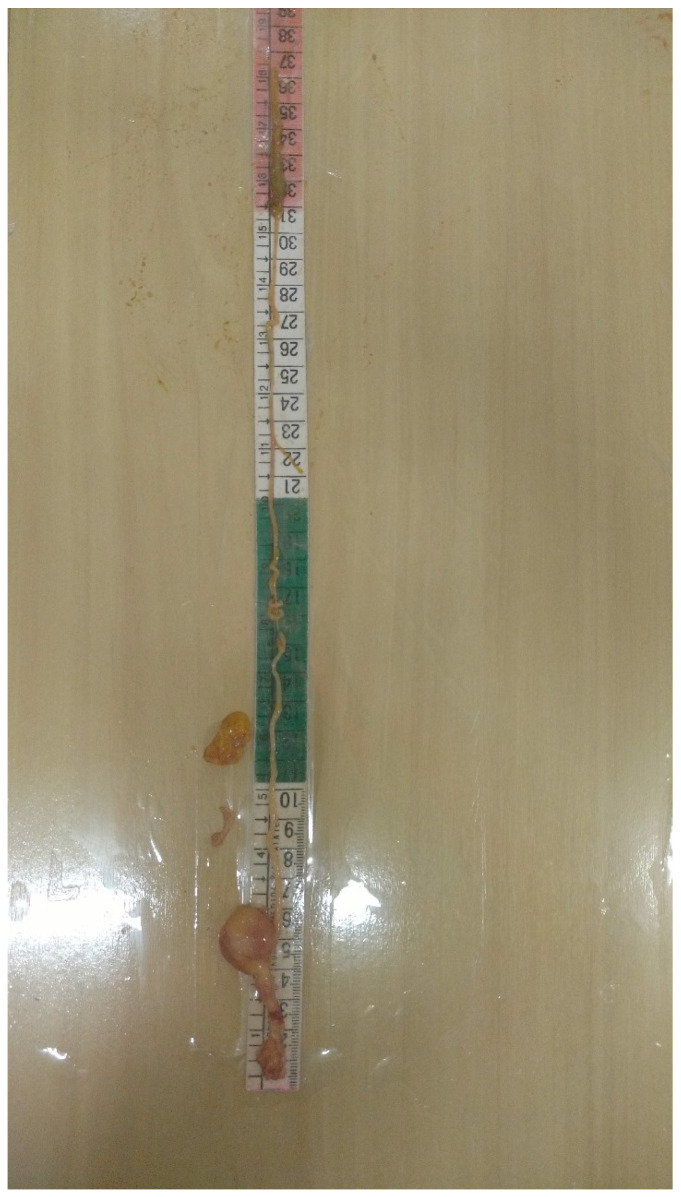
Demonstration of measuring the gastrointestinal tract of broiler chicks incubated at low temperature with different monochromatic lights.

At necropsy, the viscera (small intestine (SI), large intestine (LI), liver and pancreas) were removed, measured and weighed following the methodology of Minafra [20]: the length from the insertion of the esophagus into the oropharynx to the communication of the LI with the cloaca. Then, the weight of the organs was measured separately: the weight of the SI, the portion comprising the end of the muscular stomach until the beginning of the caecum; the weight of the LI, represented by the weight of the caecum, colon and rectum; the weight of the liver, given by the weight of the liver without the vesicle; and the weight of the pancreas, after its separation from the duodenal loop (Figure 2). Organ weights were expressed in relation to body weight without the yolk residue (organ weight × 100/body weight without the yolk residue).

For the serum biochemical evaluation, the blood of the euthanized animals was collected and the samples were identified and processed according to the methodology of Minafra et al. [21], then centrifuged at 6000 rpm for 10 min. After separation of the serum, it was immediately frozen, and the contents of calcium (Cal), phosphorus (P), total protein (TP), triglycerides (Trig), cholesterol (Col), glutamic oxalacetic transaminase (AST), glutamic pyruvate transaminase (ALT), amylase (AM), urea (U) and glucose (Glu) were evaluated. All analyses on blood plasma were performed using commercial kits from the Labtest brand with their respective recommended procedures, using colorimetric methodology.

### 2.3. Statistical Analysis

The percentage of hatching was calculated in relation to total eggs incubated. Analysis of variance of chick weight, yolk sac weight, chick weight with and without yolk sac, chick relative body weight, weight loss, biometrics of the gastrointestinal tract and biochemistry of blood and liver were performed with the aov function, considering treatment and tray as fixed effects, as well as the interaction between them. The *p*-values were determined by ANOVA function of the package car. The Shapiro–Wilk Test was used to assess whether the data distribution was similar to a normal distribution. The means were estimated by the emmeans function of the package emmeans [22], using the Tukey test to compare means.

The analysis of variance of egg weights was performed with the lmer function, considering treatment and tray as fixed effects, as well as the interaction between them, and the egg as a random effect. The *p*-values were determined by the ANOVA function of the package car, and the means were estimated by the emmeans function of the package emmeans [22], using the Tukey test to compare means.

Logistic regression, in the analysis of variance of the probability of the embryos surviving and the hatchability, was performed with the glm function, considering treatment and tray as fixed effects, as well as the interaction between them, with the egg as a random effect. The *p*-values were determined by the ANOVA function of the package car, and the means were estimated by the emmeans function of the package emmeans [22], using the Tukey–Kramer test to compare means. All analyses were performed using the computer software R version 4.3.3 (R Core Team, 2021).

## 3. Results

No effect (*p* > 0.05) was observed for the weight and weight loss of eggs incubated without and with white and red monochromatic light (Table 1).

An effect (*p* < 0.05) can be observed on the thermographic temperatures of the eggs with the use of white monochromatic light, which presented the highest temperature (MAX 36.1 °C; MIN 33.8 °C and average 35.3 °C) compared to other treatments (Table 2).

No differences were observed between the probabilities of survival of broilers at the beginning of artificial incubation, but from the seventh day on, a lower probability of survival is observed in red light (0.916) compared to no light and white light (both with 0.944). From the 14th day of incubation until hatching, eggs survive longer in the incubators without light and with white light, compared to the incubator with red light (750 nm) (Table 3).

No effect (*p* < 0.05) was observed for chick weight (CW), yolk sac weight (YSW), chick weight without yolk sac (CWWYS) and chick relative body weight (CRBW) with the use of monochrome lights in the artificial incubation process (Table 4).

No effect (*p* > 0.05) was observed for biometry of the gastrointestinal tract on the weight variables of the gastrointestinal tract (GITW), small intestine (SI), large intestine (LI), liver, pancreas and yolk sac with the use of monochromatic lights and at different incubation heights (upper, middle and lower tray) in the artificial incubation process (Table 5).

Table 6 shows the unfolding of the interaction between lighting and tray of the GITW (*p* = 0.0026). Under white light, the lower tray had a higher relative weight in the gastrointestinal tract compared to the upper and median trays, and in the lower tray, the use of color-independent lighting increased the relative weight of the gastrointestinal tract compared to no lighting.

No effect (*p* < 0.05) of lighting or tray height was observed for calcium (Ca), total protein (TP), triglycerides (Trig), glutamate oxaloacetate transaminase (AST) and glutamate pyruvate transaminase (ALT) (Table 7). However, differences were observed (*p* < 0.05) for phosphorus (P) and cholesterol (Col) and interaction (*p* < 0.05) between lighting and tray on the variables amylase (AM), glucose (Glu) and urea (U).

It is noteworthy that under white and red monochrome lights serum cholesterol and glucose levels increased, while amylase values decreased. Serum levels of phosphorus and urea were lower in chicks that received red monochromatic light.

At unfolding (Table 8), the levels of triglycerides increased in the incubators with white and red lighting in the median tray, amylase levels increased in the incubator with white lighting and decreased in the incubator with red lighting in the median tray, and amylase levels decreased in the incubators with white and red lighting over the bottom tray.

Urea values decreased in the incubators with white and red lighting in the upper tray, increased in the incubator with red lighting in the middle tray, and increased in the incubator with white lighting in the lower tray, in relation to the other treatments (Table 8). Glucose increased in the incubators with white and red lighting in the top tray.

No effect of tray height was observed on triglyceride and glucose levels for the incubator without lighting and urea levels were lower in chicks from the median tray, an influence of the absence of light and the use of white light.

## 4. Discussion

In the present study, brown-shell eggs were used, which differs from other studies found in the literature, where white-shell eggs are mostly used. The differences found may be related to the light absorption capacity of different eggshells, since eggshells are a critical factor that influences the incubation performance of eggs [15,23]. As in the present study, the color of the eggshell was brown, so the filtration capacity of the lights may have been different.

The light-absorbing capacity of the eggshell is related to the shell’s color, strength and thickness. Generally, brown eggs have larger shell thickness, on average 0.37 mm [24], while the light-pink shells have an average thickness of 0.32 mm in the intermediate period of egg laying, which affects the light absorption capacity [25].

In broilers, Archer [26] suggested that white light from LEDs is filtered so that the wavelengths of the light are red-shifted, while red and green lights are not filtered or shifted that way. The different transparency of the eggshell for different wavelengths of light can be environmentally important, since different colors of the shell can selectively filter the light to which the embryos are exposed. Eggshell pigments admit penetration or block different wavelengths of the light spectrum [27]. However, considering the particularities of each species of birds, the effects of light during the incubation period may be different [28]. Thus, more studies are needed to understand the transmission of light waves in different colors and species of birds.

Incubation temperature is the most important factor during embryo development [4]. Thus, the eggshell temperature has been used as an indicator of embryonic temperature. According to Meijerhof and Van Beek [29] and French [30], the eggshell temperature deviates around 0.1 to 0.2 °C from the embryo temperature.

Different eggshell temperatures were compared by Lourens et al. [31], in which embryos exposed to a temperature of 37.8 °C throughout incubation showed better hatchability rates (88.1%) and embryonic development when compared to embryos submitted to a high temperature (38.9 °C), which obtained 86.2% hatchability.

Willemsen et al. [32] observed reduced hatchability (74.2%) in treatment with high shell temperature (40.6 °C) when compared to a temperature of 37.8 °C (93.1%), possibly caused by increased final embryonic mortality (13.8% vs. 2.5%, respectively). Van der Pol et al. [33] also found lower hatching values (78.4%) and organ development in embryos exposed to high temperatures (38.6 °C) when compared to the control group (95%).

High or low incubation temperatures can influence embryogenesis, affect blood biochemical levels and chick quality and, consequently, productive performance during breeding [34]. The three eggshell temperatures were 34.6 °C (low), 37.6 °C (control), or 40.6 °C (high) during incubation days [32].

According to data from our study, the minimum temperature of the eggs is low (34.1 °C treatment without lighting; 35.1 °C treatment with white lighting and 34.3 °C treatment with red lighting) in all treatments with and without lighting, so the low temperature provided thermal stress in the embryonic development of the chicks. The temperature data of the eggs were determined using an infrared thermographic camera, while in the studies mentioned above it was determined by a thermohygrometer coupled to the eggshell, so values may be slightly different because of the technology employed.

Regarding YFBM (yolk free body mass), this is a quality parameter that represents the body weight of the chick without the weight of the residual yolk sac. Lower yolk sac weight and higher YFBM are desirable, since they indicate ideal environmental conditions in incubators and hatcheries, in addition to the embryo’s ability to use yolk components for structural formation [9]. Joseph et al. [35], while studying the effect of high incubation temperatures (39.5 °C) on chick weight, observed a reduction in body weight (39 g) and lower YFBM (34.7 g) compared to a temperature of 37.8 °C, where the results were 40 g of body weight and 35.9 g of YFBM. However, in the present study, no differences were observed for chick weight, yolk sac weight, chick weight without yolk sac and chick relative body weight with the use of monochrome lights in the artificial incubation process.

Relative humidity should also be controlled within the recommended limit, as its deviations affect the quality of the newborn chick [36]. When the relative humidity (RH) is above ideal during incubation, the chick’s weight increases, as excess water is incorporated into the embryonic tissues, impairing its initial performance [37]. Thus, the relative humidity found in the present study is considered high (maximum RH was 72% and minimum RH was 68%), but no significant difference was found for egg weight and weight loss in this study.

The loss of egg mass is directly linked to water loss, and, under normal conditions, the egg loses 13 to 15% of its mass between the day of laying and the day of internal pecking [30]. The egg starts to lose water after oviposition. For optimal hatchability, egg weight loss is expected to be close to 6.5–12.0% [38,39]. Water loss is also an indicator of the embryo’s metabolic rate and a high metabolic rate accelerates water loss [40]. In the present experiment, weight loss is within the normal range, with an overall average of 10.33%, and not impairing the embryonic development of the eggs.

When eggs are exposed to periodic photo lighting, melatonin secretion is stimulated and begins to establish a circadian rhythm in the pineal gland from the sixteenth day of embryonic development [41]. The provision of photoperiod during incubation can stimulate pineal melatonin secretion and regulate growth hormone synthesis. However, the increase in overall embryo weight as well as muscle weight was also found when there was continuous light exposure in chicken embryos [42,43]. Such results indicated that muscle growth may depend on light exposure, but it is not associated only with circadian rhythms commanded by the photoperiod. The wavelength and intensity of light can influence the amount of light that can pass through the eggshell and reach the embryo. Light intensity may be one of the main factors affecting mitosis in the mesoderm of the neural crest during the early stage of embryonic development [44]. However, in the present study, no difference was found for increased embryo weight.

Increase in hatching may be attributed to positive changes in hormones related to hatching, especially thyroid and corticosterone, in response to lighting treatments. Such improvement may also be due to the positive impact on the rate of embryo development, and the study by Ghatpande et al. [45] suggested that light during incubation increases the rate of embryonic development. Furthermore, it may also be due to the change in the rhythm of melatonin in the embryos provided by light during incubation [46]. No improvement in hatching was observed in the current study.

Geng et al. [47] found that birds which did not receive light stimulus during the 21 days of incubation showed the highest percentage of hatching, demonstrating that instead of promoting benefits, the exposure of embryos to green light resulted in hatchability impairment. However, in the present study, the lights used were white and red, with no effect on hatching between treatments.

Serum biochemical indices may partially reflect the metabolism and health status of the organism, especially serum immunological and antioxidant indices, which indirectly reflect the animal’s health status [47]. Responses to stressors are evidenced first at the biochemical level, followed by physiological responses and, finally, manifested at morphological level. Thus, changes in all these levels are indicative of stress and, therefore, parameters such as plasma glucose are important physiological indicators of stress levels [48]. In the present study, differences were observed in biochemical parameters (Calcium, Phosphorus, Cholesterol, Amylase, Glucose, Urea and TGP), which may be explained by low temperature stress in conjunction with the use of monochromatic lights in eggs.

Heat stress causes multiple changes in the neuroendocrine physiology of birds. Several studies report continuous activation of the hypothalamic–pituitary–adrenal axis, which promotes increased circulating levels of corticosterone, resulting in greater protein catabolism, hyperglycemia, and immunosuppression and increased susceptibility to infections [49,50].

Studies reveal that thermal manipulation during incubation, in the development periods of the hypothalamic–pituitary–thyroid and hypothalamic–pituitary–adrenal axes of the embryo, promotes epigenetic and metabolic changes, which allow birds to adapt to high and low environmental temperatures [51,52]. Such changes are in the stress regulatory pathways, decreasing metabolic rate and increasing the expression of pro-angiogenic genes in muscle. This process enhances the vasomotor response and, consequently, heat loss as well, as it increases the expression of anti-apoptotic genes, preserving the integrity of cells during thermal challenge after hatching [53].

Guest et al. [54] show increases and decreases in several hormones and compounds whose metabolisms are directly impaired in each phase of stress. In the alert phase, there is an increase in noradrenaline and adrenaline and in glucose, and therefore, in the individual’s heartbeat, in addition to vasoconstriction and mydriasis in individuals. In the exhaustion phase, an increase was observed for levels of fatty acids, such as triglycerides and cholesterol, for glycerol and leukocytes. Such an increase can directly affect the physiology, causing increased heart rate, vasoconstriction, inhibition of insulin, and increase in blood glucose, cholesterol and triglycerides.

Photostimulation with green monochromatic light during the late phase of incubation (between ED18 and ED20), which is considered a critical phase in embryonic development, increases the activity of the somatotropic axis at the level of the positive control; thus, a critical period for photostimulation of broiler embryos can be considered. The critical period is defined by several physiological mechanisms, including the transition to pulmonary respiration and increased hepatic gluconeogenesis to increase glucose levels and provide sufficient energy for the embryo to hatch [55], with this pattern presented in the white and red light used in the present study.

According to Artacho et al. [56], the physiological state can be reflected according to plasma metabolites, which are intermediate products of metabolism. All physiological and metabolic mechanisms carried out during embryogenesis, hatching and post-hatching can be directly affected by temperature at incubation. Studies show that situations of high and low temperatures and/or low oxygen availability can interfere with the physiology and metabolism of the chicken embryo and, consequently, promote harmful effects on embryo development and survival [31,57,58].

At the beginning of incubation, the oxygen support of the embryo is extremely limited due to the immature state of the vascular system. Thus, anaerobic glycolysis becomes active, but in a restricted way, as glucose levels are minimal at this stage [59]. In the final third of incubation, the embryo also uses alternative routes, such as gluconeogenesis for glucose production, which will be stored as liver and muscle glycogen for use in the final moments of incubation [60].

The hatching process requires high energy demand and the fatty acids can no longer efficiently supply all the necessary fuel. Glucose is released from glycogen and the embryo performs anaerobic glycolysis until external pecking, increasing the circulating lactate [61]. Hoiby et al. [62] stated that after internal pecking, the embryo comes into contact with the tube, and the supply of oxygen for the metabolism is resumed. The amount of lactate decreases and the catabolism of fatty acids starts the energy supply again. In the period shortly after the hatching of chicks, the synthesis of glucose from the oxidation of fatty acids is intensified [63].

Regarding the storage of glucose in the animal organism, this occurs through the glycogenic route with the formation of glycogen, which is stored primarily in the liver, in addition to the membrane of the yolk sac, breast muscle and intestines [64,65].

Glycogen is the energy reserve polysaccharide of animal organisms and it stores glucose in a readily mobilizable form. Therefore, in the absence of plasma glucose, hepatic and renal glycogen is mobilized for glucose release by glycogenolysis. In the case of muscle glycogen, degradation occurs in response to tissue energy expenditure. When there are limiting levels of glycogen, some tissues produce glucose from amino acids of the residual proteins. The main storages of this compound are found in the liver and skeletal muscle. In the liver, glycogen plays a role in maintaining the concentration of glucose in the blood, especially in the early stages of fasting, while muscle glycogen acts as a fuel reserve to synthesize ATP molecules [66].

Glycogen reserves in the final incubation period are abundantly mobilized, indicating the essentiality of glucose in the artificial incubation of chicken eggs [67]. According to Moran Jr [59], the storage of hepatic glycogen reserves is of paramount importance, as it is a preparation for the hatching process. One mechanism to conserve glycogen is the gluconeogenesis process by the liver from non-glycosidic compounds such as glycerol and amino acids [68].

During the first days of incubation, the embryo has low oxygen supply due to the immaturity of blood cells and the vascular chorionic system [69]. According to Moran Jr [59], the embryonic compensatory mechanism in those situations is to perform anaerobic glycolysis, and therefore, the lactate increase is pronounced and considered normal in those stages. In addition, glycogen is degraded to release glucose molecules that will be oxidized to pyruvate. Pyruvate is transformed into lactate in anaerobiosis [32,70].

Harms and Harms [71] related high plasma lactate concentrations in response to stressful stimuli. Referring to the hatching process, a large amount of energy is required for the rotational movements of the body and breaking of the shell. Glycogenic stores are depressed and a low glucose concentration forces the embryo to mobilize body protein. At the end of incubation, the metabolism is increased to obtain glucose and, due to the lack of oxygen in those last stages, the embryo cannot produce energy from lipid metabolism, becoming dependent on the gluconeogenic pathway [59].

Then, the anaerobic production of lactate is intensified, and this is converted to glucose. Accordingly, there is a progression of lactate levels at high and low incubation temperatures as muscle activity is high and oxygen availability is low during the hatching process. The pyruvate formed during anaerobic glycolysis for lactate formation increases the blood hydrogen proton and is buffered by bicarbonate (HCO_3_), avoiding a reduction in pH [55].

Although amylase is not directly related to the gluconeogenic pathway, it is noteworthy that glucose is one of the products of starch digestion by amylase. The resulting glucose can be used by the body’s cells as an energy source, and the excess is stored as glycogen in the liver and muscles, to be used later. In situations of low glucose availability, such as prolonged fasts, gluconeogenesis is activated to maintain blood glucose levels. Amylase is an enzyme present in the body whose main function is to break down starch into smaller sugar molecules, such as glucose [68]. However, no direct relationship between amylase and the gluconeogenic pathway could be detected in this study.

The serum decrease in urea is not indicative of kidney injury, because it originates from the hepatic metabolism of nitrogenous compounds. It can also be an indicator of the use of gluconeogenesis as an alternative route for energy production [69]. In this study, the increased use of gluconeogenesis was due to increased glucose and decreased urea for lighting treatments.

During the transition from the pre-eclosion phase to the post-eclosion phase, metabolic heat is rapidly produced by the rapid rate of fatty acid oxidation [59]. Yalcin et al. [70] reported changes in fatty acid composition and concentrations in high-yield broilers when eggs were incubated at high and low temperatures. This probably occurs as a cell protection mechanism, since many of these lipids are involved in the permeability and structure of developing cells.

Connor et al. [71] found that 98% of the chicks’ cholesterol at hatching comes from the yolk. Thus, the increase in the cholesterol rate promoted by the low incubation temperature must have resulted from greater use of the yolk sac recorded for those chicks. The results corroborate those found by Willemsen et al. [32], who also recorded higher cholesterol values for egg chicks incubated at a lower-than-usual temperature. The white and red lights followed this profile for cholesterol.

Calcium is the mineral with the highest concentration in the bird’s organism, being a relevant part in the formation of eggs and eggshells, in addition to participating in many important biochemical reactions [72]. Phosphorus, like calcium, is an important part of bone formation and participates in the regulation of acid–base metabolism and energy production [73]. This last function, due to the presence of large amounts of phosphate compounds in red blood cells, gives birds a reduction in oxygen affinity, offering an advantage for the regulation of oxygen transport [74]. There is interdependence between calcium and phosphorus values, so that the deficiency or excess of one of them can impair the absorption or use of the other [75].

Glutamate pyruvate transaminase is an enzyme present in high concentration in the kidney, heart, skeletal muscles, liver and lung, and therefore the interpretation of its value in poultry liver diseases is controversial [76,77]. Glutamate oxaloacetate transaminase is widely distributed in poultry and is present in high concentration in various organs and tissues, mainly heart, liver, skeletal muscles, kidney and brain. However, the enzyme’s distribution differs among avian species. Thus, it cannot be considered as a hepato-specific enzyme, as it also indicates muscular sensibility [78].

The metabolites of AST and ALT provide information about the functional capacity of the liver that is involved in a given metabolic pathway. AST is the most sensitive indicator of liver disease in poultry [79]. Liver damage causes the release of AST or ALT enzymes into the bloodstream. Hassan et al. [80] mentioned that monochromatic lights in broiler chicks did not alter liver enzymes (AST and ALT) in a healthy batch. Olanrewaju et al. [81] reported that the use of two-color temperatures of LED lamps did not influence the plasma concentrations of AST and ALT in broilers. The result differs from the present study in terms of ALT in the blood of chicks in the first hours of birth submitted to monochromatic white and red lighting and to no light, in which ALT was not detected in the interaction.

## 5. Conclusions

The use of red and white monochromatic light in the artificial incubation process for brown-colored eggs is not recommended. The post-hatching phase promoted dysregulation of metabolism over the blood biochemical profile in an attempt to control the wavelength differentiation of traditional incubation.

## Figures and Tables

**Table 1:** Weight and weight loss of broiler eggs per days of incubation, submitted to incubators without lighting and with white and red monochrome light.

PODI * (g)	Average						*p*-Value			CV ^1^
	Lighting			Tray			Lighting	Tray	LxT	
	No Light	White Light	Red Light	High	Median	Low				
7°	0.066	0.067	0.067	0.066	0.067	0.067	0.0682	0.0686	0.0673	6.05
14°	0.064	0.064	0.065	0.064	0.064	0.065	0.1270	0.1948	0.3709	6.05
18°	0.062	0.063	0.064	0.062	0.063	0.064	0.0981	0.0650	0.0632	6.07
WL (%)	9.55	12.13	9.32	10.57	10.51	9.92	0.5004	0.8372	0.7787	10.01

* Egg weight in grams per day of incubation; WL (%): egg weight loss in percentage. ^1^ Coefficient of variation. LxT: interaction between lighting and trays.

**Table 2:** Thermographic temperature per day of broiler eggs, in the artificial incubation period, submitted to incubators without lighting and with white and red monochromatic light.

Temp °C	Average						*p*-Value			CV ^1^
	Lighting			Tray			Lighting	Tray	LxT	
	No Light	White Light	Red Light	High	Median	Low				
MAX	35.3 ^b^	36.1 ^a^	35.3 ^b^	35.6	35.7	35.4	0.0021	0.2204	0.842	0.97
MIN	32.7 ^b^	33.8 ^a^	32.7 ^b^	33.8 ^a^	33.7 ^a^	31.7 ^b^	0.0004	0.0001	0.201	1.51
Average	34.4 ^b^	35.3 ^a^	34.4 ^b^	34.9 ^a^	35.0 ^a^	34.2 ^b^	0.0004	0.0002	0.641	1.02

Temp: temperature; MAX: maximum temperature; MIN: minimum temperature. ^1^ Coefficient of variation. LxT: interaction between lighting and trays. Different letters on the same line are different according to Tukey’s test.

**Table 3:** Probability of broilers surviving in the artificial incubation period, submitted to incubators without lighting, and with white and red monochromatic light.

SODI * (%)	Processing		
	No Light	White Light	Red Light
1°	1.000	1.000	1.000
7	0.944	0.944	0.916
14°	0.916	0.906	0.879
18°	0.916	0.907	0.879
Hatch	0.631	0.573	0.524

* Survival of embryos by days of incubation.

**Table 4:** Chick weight, yolk sac weight, weight without yolk sac, and relative body weight of chicks in grams submitted to incubators without lighting and with white and red monochrome light.

Variable *	Average						*p*-Value			CV ^1^
	Lighting			Tray			Lighting	Tray	LxT	
	No Light	White Light	Red Light	High	Median	Low				
CW (g)	50.2	49.3	49.9	50.7	49.0	49.7	0.7412	0.2528	0.3502	6.15
YSW (g)	14.6	14.5	14.9	15.2	14.7	14.2	0.9404	0.8208	0.9546	12.05
WWYS (g)	35.6	34.8	35.0	35.6	34.3	35.5	0.6166	0.3699	0.1669	7.82
CRBW (g)	82.6	77.4	74.2	79.8	78.2	76.3	0.1119	0.2362	0.5136	7.66

* CW: Chick weight; YSW: yolk sac weight; WWYS: weight without yolk sac; CRBW: chick relative body weight. ^1^ Coefficient of variation. LxT: interaction between lighting and trays.

**Table 5:** Biometrics of the gastrointestinal tract of broilers submitted to incubators without lighting and with white and red monochromatic light.

Variable *	Average						*p*-Value			CV ^1^
	Lighting			Tray			Lighting	Tray	LxT	
	No Light	White Light	Red Light	High	Median	Low				
GITW (g)	9.36	11.30	10.60	9.68 ^b^	10.10 ^b^	11.48 ^a^	0.1138	0.0001	0.0026	14.09
SI (g)	1.14	1.53	1.44	1.35	1.25	1.52	0.3140	0.0925	0.2302	23.28
LI (g)	0.713	1.199	0.792	0.811	0.914	0.980	0.1656	0.2906	0.1216	33.91
Liver (g)	1.61	1.55	1.65	1.51	1.60	1.70	0.2996	0.3439	0.1204	9.96
Pancreas (g)	0.0546	0.0869	0.0622	0.0826	0.0492	0.0719	0.3061	0.0604	0.3242	49.55
YOLK SAC (g)	99.5	78.5	86.3	98.6	90.7	75.0	0.7151	0.2154	0.6143	29.65
GITYOLKSAC (g)	158.0	136.0	143.0	160.0	146.0	130.0	0.7266	0.0956	0.4864	21.18

* GITRW (g): Relative weight of the gastrointestinal tract; SI (g): small intestine and LI (g): large intestine; YOLK SAC (g): yolk sac; YOLKSACGIT: yolk sac and gastrointestinal tract. ^1^ LxT coefficient of variation: interaction between lighting and trays. Different letters on the same line are different according to Tukey’s test.

**Table 6:** Unfolding of the lighting inside the tray and unfolding of the tray within the lighting: the weight of the gastrointestinal tract that was significant in the interaction between lighting and the trays of broiler eggs submitted to incubators without lighting and with white and red monochromatic light.

GITW			
Lighting	Tray		
	B1	B2	B3
No light	9.24 ^Aa^	9.63 ^Aa^	9.21 ^Ba^
White light	9.22 ^Ab^	10.86 ^Ab^	12.93 ^Aa^
Red Light	10.56 ^Aa^	9.80 ^Aa^	11.43 ^Aa^

B1: upper tray; B2: medium tray; B3: lower tray; GITW: gastrointestinal tract weight. Uppercase letters in the same column are different according to Tukey’s test (unfolding of the lighting inside the trays). Lowercase letters on the same line are different according to Tukey’s test (unfolding of the trays within the lighting).

**Table 7:** Blood biochemical profile of broilers submitted to incubators without lighting and with white and red monochromatic light.

Variable *	Average						*p*-Value			CV ^1^
	Lighting			Tray			Lighting	Tray	LxT	
	No Light	White Light	Red Light	High	Median	Low				
Ca (mg/dL)	12.0	11.9	10.4	12.1	10.9	11.3	0.4693	0.6397	0.0564	25.38
P (mg/dL)	9.12 ^ab^	9.37 ^a^	8.08 ^b^	9.45	9.10	8.01	0.0108	0.7622	0.0682	10.77
TP (g/dL)	2.29	2.55	2,82	2.40	2.66	2.61	0.2899	0.9051	0.5295	16.85
Chol (mg/dL)	97.4 ^b^	115.5 ^a^	109.7 ^ab^	96.5 ^b^	113.9 ^a^	112.3 ^a^	0.0424	0.0048	0.0585	11.55
Trig (mg/dL)	93.8	134.2	146.0	92.1 ^b^	163.9 ^a^	117.6 ^a^	0.9953	0.0024	0.0083	20.87
AM (mg/dL)	611.0 ^a^	590.0 ^b^	563.0 ^c^	597.0 ^a^	596.0 ^a^	571.0 ^b^	0.0001	0.0001	0.0001	1.21
Glu (mg/dL)	120.0 ^b^	125.0 ^a^	123.0 ^a^	120.0	123.0	125.0	0.0001	0.9482	0.0004	2.24
Urea (mg/dl)	33.1 ^a^	32.3 ^ab^	31.7 ^b^	31.7 ^b^	33.6 ^a^	31.8 ^b^	0.0001	0.0001	0.0001	4.79
AST (mg/dL)	158.0	166.0	156.0	166.0	163.0	151.0	0.8304	0.6853	0.0983	11.61
ALT (mg/dL)	47.7	51.0	49.0	50.2	51.6	45.8	0.6429	0.6751	0.3288	21.38

* Ca (mg/dL): Calcium; P (mg/dL): Phosphorus; PT (g/dL): total proteins; Chol (mg/dL): Cholesterol; Trig (mg/dL): Triglycerides; AST (mg/dL): Glutamate-oxaloacetate transaminase; ALT (mg/dL): Glutamate-pyruvate transaminase; AM (mg/dL): Amylase; Glu (mg/dL): Glucose. ^1^ Coefficient of variation. LxT: interaction between lighting and trays. Different letters on the same line are different according to Tukey’s test.

**Table 8:** Unfolding of the lighting within the tray and unfolding of the tray within the lighting: the biochemical profile variables that were significant in the interaction between lighting and egg trays of broilers submitted to incubators without lighting and with white and red monochromatic light.

Lighting	Tray		
	B1	B2	B3
Triglycerides			
No light	92.4 ^Aa^	94.8 ^Ba^	92.8 ^Aa^
White light	91.0 ^Ab^	178.8 ^Aa^	132.7 ^Aab^
Red Light	93.0 ^Ab^	218.0 ^Aa^	127.2 ^Ab^
Amylase			
No light	617 ^Aa^	603 ^Ba^	612 ^Aa^
White light	577 ^Ab^	620 ^Aa^	573 ^Bb^
Red Light	598 ^Aa^	564 ^Ca^	528 ^Ca^
Urea			
No light	41.6 ^Aa^	30.8 ^Bb^	26.9 ^Bc^
White light	20.1 ^Cc^	32.3 ^Bb^	44.5 ^Aa^
Red Light	33.5 ^Bb^	37.6 ^Aa^	24.1 ^Bc^
Glucose			
No light	112.0 ^Ba^	120.0 ^Aa^	127.0 ^Aa^
White light	125.0 ^Aa^	125.0 ^Aa^	124.0 ^Aa^
Red Light	123.0 ^Aa^	125.0 ^Aa^	123.0 ^Aa^

T1: top tray; T2: mid tray; T3: bottom tray. Uppercase letters in the same column are different according to Tukey’s test (unfolding of the lighting inside the trays). Lowercase letters on the same line are different according to Tukey’s test (unfolding of the trays within the lighting).

## Data Availability

Data are contained within the article.
